# Supplementary material for: Assessment of health staff’s proficiency and quality of key malaria indicators in rural district of Ghana
Source: PLoS One. 2022 Oct 27;17(10):e0274700. doi: 10.1371/journal.pone.0274700 (PMC9612565; doi:10.1371/journal.pone.0274700)
Supplement: Attachment — Submitted filename: Response to Reviewers Comments (26.07.2022).pdf [file pone.0274700.s005.pdf]

26 July 2022

Editor-in-Chief
PLOS ONE

Dear Sir/Madam,

Re: Responses to Reviewers and Editor's Comments

On behalf of my colleagues, I am submitting responses to Reviewers and Editor's comments raised in our article "*Assessment of health staffs' proficiency and quality of key malaria indicators in Rural District of Ghana".*

The following areas of concern raised by the reviewers and Editor with responses (highlighted) as detailed below:

Discussion on specific area of improvement

1. Ethical consideration: pls mention the name of institution that the approval was issued, together with approval number. For example: Ethical approval was obtained from xxxx institution (approval number xxxx).
   - ✓ *Thank you for your feedback. The issues raised have been resolved. That is* "Ethical approval was obtained from the Cape Coast Teaching Hospital Ethical Review Committee (approval number: CCTHERC/EC/2020/045)" *[see lines 222-223]*

Results

2. Majority of the description in the text for the table and figures are still not revised yet. Pls describe and interpret the key findings ONLY in the text, not to duplicate information with the table and text. Pls revise all text in the result session and check the use of past-tense (verb) consistently in the result session in terms of English language editing.
   For example: "*The specialization of respondents included: Doctor (2.0%), Pharmacist (2.0%),*
   252 Physician Assistant (2.0%), Nurses/Midwives (42.0%), Technical Officers (14.0%), Dispensing Technician (2.0%),253 Health Information Officers/ Biostatisticians (8.0%), and Lab scientist/Technician (6.0%) as shown in Table 3." [need to revise, this is just description of the result and duplicated with the information from the table. This is not the interpretation of the key findings.]
   The above sentence should be described as followed as the example. *"The majority of the respondents were nurses/ midwives (42.0%), followed by the technical officers (14.0%) and health information officers/ biostatisticians (8.0%). Doctors and physician assistants were contributed only 2% of the respondents respectively."*
   - ✓ *Thank you for your feedback. We have revised the results section and corrected all typo and grammatical errors that we could find. Furthermore, we had to rephrase certain constructions and dictions that seemed out of place [see lines 237-347]. The remaining tables 4-7 and their corresponding texts were not considered because we had already revised same, and for fear of run counter to whatever we wanted to communicate*.

Discussion

3. Thank you for revising, yet, discussion is still needed to improve more concise in description in writing. I would recommend to consult/ take the proper English language editing service for academic writing.
   - ✓ *Thank you for your feedback. We have corrected all typo and grammatical errors that we could find. Furthermore, we had to rephrase certain constructions and dictions that seemed out of place [see lines 410-513].*

Thank you for your reconsideration and re-evaluation prior to consideration for publication. My colleagues and I appreciate your time and effort and look forward to hearing from you.

Sincerely,

Richard Okyere Boadu (PhD)
Department of Health Information Management
School of Allied Health Sciences
College of Health and Allied Health Sciences
University of Cape Coast
Cape Coast, Ghana
richard.boadu@ucc.edu.gh
