# Supplementary material for: Assessment of health staff’s proficiency and quality of key malaria indicators in rural district of Ghana
Source: PLoS One. 2022 Oct 27;17(10):e0274700. doi: 10.1371/journal.pone.0274700 (PMC9612565; doi:10.1371/journal.pone.0274700)
Supplement: Attachment — Submitted filename: Reviewer comment_PONE-D-22-00045_22Apr2022.docx [file pone.0274700.s002.docx]

**Reviewer comment for PLOS ONE**

Date of review: 13 Apr 2022

Manuscript Number: PONE-D-22-00045

Article Type: Research Article

Full Title: Assessment of health staff’s proficiency and quality of key malaria indicators in Rural District of Ghana

Corresponding Author: Richard Okyere Boadu, University of Cape Coast, Cape Coast, GHANA

**Reviewer comment**

Summary of the research and overall impression

This manuscript presented to identify the health staff’s proficiency and quality of data on key malaria indicators in selected rural district of Ghana. Overall, the study will benefit to the national malaria control program to understand the requirement and extent/ capacity of local health staff at the health facilities level. The findings will provide input for the national program to enhance and strengthen the data quality assurance which is one of the most important factors for malaria elimination.

Technically, data presentation and data analysis, methodology is good, however the major improvement is needed for presenting in text in the results session, discussion, conclusion and recommendation.

English language is strongly recommended for academic publication, in terms of using the correct tense (in verb), academic writing and language of data presentation throughout the manuscript.

Discussion on specific area of improvement

Abstract

1. The information presented in the abstract is concise. However, the last two sentences of **result** session are not clear. Please edit for clear statement.
2. In **conclusion**, it is needed to revise mentioning – what we conclude from the key findings and what is the major implication of the findings for the action plan or for the future.
3. Language editing is required for abstract. Academic writing should be edited and the past-tense (verb) should be used.

Introduction

4. Suggest to add “*Global Technical Strategy for Malaria 2016–2030*” in the list of reference.
1. Pg-2, paragraph-2, line-6: Please clarify “*These facilities are mandated to use these standard tools to collect and present data*”.
2. Pg-2, paragraph-3, line-6: Please expel “MICS”.
3. Pg-2, paragraph-3, line-8: “*28414 cases were confirmed*” please mention confirmed by what method of diagnosis. (eg. confirmed positive by RDT?)
4. Pg-2, paragraph-3, line-12: Please correct the spelling of LLIN = “Long-lasting **insecticidal** nets”
5. Pg-2, paragraph-4, line-10: *“…..this is not different from other studies in Ghana and* ……” please add the reference for this statement. The current reference [24] is from Uganda representing for “*other sub-Saharan countries*”.
6. Lastly, please add more information for why this study is important to conduct (justification) and how this study can provide supplementary information or input to the health system, in addition to other studies about HMIS conducted in the Ghana.

Methods

7. Study area: please include total number of populations, male: female ratio, population density, total populations that one health facility covers, number of health staff (eg. doctor: patient ratio, etc)
8. Selection of malaria indicator: please mention what type of malaria test was used for diagnosis and confirmed malaria positive (eg. RDT, PCR, microscopy?)

Results

9. Please add the data table and description about the background characteristics of study sites, study populations, etc. (eg. sample size by each study arm, by study sites, total tested, by time point etc)
10. Expressing each indicator is good and comprehensive, but the description in the text for the table is too detail. Suggest only key findings should be presented in the text, not to duplicate information with the table and text. Please re-write the results text.
11. Table 2. The “total” row is not necessary. Please delete. Please re-order the category of staff according to the number from the largest to the smallest.
12. Chart 4. Should explain and describe how the data in chart 4 was calculated, in terms of “summative” – which the term is only appeared and described in the discussion (one time) and conclusion (one time).

Discussion and conclusions

13. Discussion: Please add more literature to compare and describe what are the difference in lessons learnt with other studies. The comparative of writing about other literature is weak in the discussion session.
14. Please discuss the point that VF values for the indicators (summative), except OPD cases treated with ACT, were within the acceptable range, but they all lied below “1” which is over-reported. Please explain and discuss for this point, which could tend to be more over-reported in the reality or other health facilities not included in the study.
15. Conclusion: pg 14, line 6, it said malaria “**eradication”.** Is it truly that the program aimed for malaria **eradication** or **elimination**? Please recheck.
16. The conclusion should be more precise and concise, touching all key findings and recommendation for program implementation. There is a duplication of mentioning about the “OPD cases treated with ACT” and suggest to combine the duplicate description. Please revise the conclusion session.
17. Recommendation must be training, refresher training and strengthen regular supervision & monitoring plan by the national program. Further research is required, but only focus on the OPD cases and ACT treatment indicator is not the key recommendation from this paper. Yes, it is the only one indicator which needs improvement and pay attention, but the recommendation should be more in general and benefit for the malaria program as an overview. Please revise the recommendation.

Recommendation

I would like to propose the **major revision** for this manuscript.
