# Supplementary material for: Assessment of health staff’s proficiency and quality of key malaria indicators in rural district of Ghana
Source: PLoS One. 2022 Oct 27;17(10):e0274700. doi: 10.1371/journal.pone.0274700 (PMC9612565; doi:10.1371/journal.pone.0274700)
Supplement: Attachment — Submitted filename: Response to Reviewers Comments (23.05.2022).docx [file pone.0274700.s003.docx]

23 May 2022

Editor-in-Chief

PLOS ONE

Dear Sir/Madam,

**Re: Responses to Reviewers and Editor’s Comments**

On behalf of my colleagues, I am submitting responses to Reviewers and Editor’s comments raised in our article “*Assessment of health staffs’ proficiency and quality of key malaria indicators in Rural District of Ghana”.*

The following areas of concern raised by the reviewers and Editor with responses (highlighted) as detailed below:

Abstract

1. The information presented in the abstract is concise. However, the last two sentences of the **result** session are not clear. Please edit for clear statement.

- *Thank you for your feedback. The sentences have been revised to read, “However, the verification factor (VF) of the overall malaria indicator shows…” [see lines 36-37]*

2. In **conclusion**, it is needed to revise mentioning – what we conclude from the key findings and what is the major implication of the findings for the action plan or for the future.

- *Thank you for your feedback. The conclusions have been revised to read, “Majority of staff had not received any training on malaria-related RHIS. Some staff do not know the correct definitions of some of MI used in malaria programmes while the majority of them could not demonstrate the correct calculations of MI. Timeliness of reporting was below the target, nevertheless, copies of data that were submitted were available and completed. There should be training, supervision and monitoring to enhance staffs’ proficiency and improved quality of MI.” [see lines 38-42]*

3. Language editing is required for abstract. Academic writing should be edited and the past-tense (verb) should be used.

- *Thank you for your feedback. Language editing has been done.*

Introduction

4. Suggest to add “*Global Technical Strategy for Malaria 2016–2030*” in the list of reference.

- *Thank you for your feedback. We have added to the reference list [see line 66]*

1. Pg-2, paragraph-2, line-6: Please clarify “*These facilities are mandated to use these standard tools to collect and present data*”.

- *Thank you for your feedback*. *The sentence has been rephrased to read, “GHS has therefore mandated the public and mission health facilities to use these registers and forms as standard tools to collect and present data” [see lines 71-72]*

2. Pg-2, paragraph-3, line-6: Please expel “MICS”.

*Thank you for your feedback*. “MICS*” is expelled from the text [see line 91]*

3. Pg-2, paragraph-3, line-8: “*28414 cases were confirmed*” please mention confirmed by what method of diagnosis. (e.g. confirmed positive by RDT?)

- *Thank you for your feedback*. *The sentence has been reconstructed to read, “For instance, about 28414 cases were confirmed positive by laboratory/RDT, …” [see line 94]*

4. Pg-2, paragraph-3, line-12: Please correct the spelling of LLIN = “Long-lasting **insecticidal** nets”

- *Thank you for your feedback. The error has been corrected [see line 97]*

5. Pg-2, paragraph-4, line-10: *“…..this is not different from other studies in Ghana and* ……” please add the reference for this statement. The current reference [24] is from Uganda representing for “*other sub-Saharan countries*”.

- *Thank you for your feedback*. *The original statement has been modified, and additional references added. The statement has been rephrased to read: “This has limited the use of malaria data to meet the reporting needs of the municipal. managers. Previous research in Ghana and other sub-Saharan African countries has highlighted issues of inaccurate reporting of malaria cases as well as treatment plans, wrongly categorizing malaria cases in registers used to collate data and misplacing data and or registers for reporting [25-28].” [see lines 107-110]*

6. Lastly, please add more information for why this study is important to conduct (justification) and how this study can provide supplementary information or input to the health system, in addition to other studies about HMIS conducted in the Ghana.

- *Thank you for your feedback*. *Additional information has been added while the sentence has been rephrased to read, “While previous attempts to address the issues of Malaria data quality has concentrated at the national level discrepancies with collated data against what pertains at district and regional levels. Little research has evaluated the contribution of facility-level staff proficiency in Malaria data quality to identify potential gaps and implement any data quality improvement intervention. This analysis is intending to contribute to filling this knowledge gap as Ghana moves towards the elimination of Malaria. This study presents findings of data quality concerning health staffs’ proficiency, timeliness, availability and data accuracy in the Sissala East MHD”. [see lines 111-116]*

Methods

7. Study area: please include the total number of populations, male: female ratio, population density, total populations that one health facility covers, number of health staff (e.g. doctor: patient ratio, etc.)

- *Thank you for your feedback. Characteristics of the facilities included in the study are shown in Table 1. [see lines 137-142]*

8. Selection of malaria indicator: please mention what type of malaria test was used for diagnosis and confirmed malaria positive (e.g. RDT, PCR, microscopy?)

- *Thank you for your feedback. Depending on the facility either microscopy or RDT or both tests were used for diagnosis and confirmed malaria positive. [see lines158-159]*

Results

9. Please add the data table and description of the background characteristics of study sites, study populations, etc. (e.g. sample size by each study arm, by study sites, totally tested, by time point etc.)

- *Thank you for your feedback. A data table and description of the background characteristics of study sites, and study populations are provided in Table 1[see lines 137-142].* *An additional table showing sample size by each study arm, by study sites, total tested, time point etc. is provided as a supplementary material.*

10. Expressing each indicator is good and comprehensive, but the description in the text for the table is too detail. Suggest only key findings should be presented in the text, not to duplicate information with the table and text. Please re-write the results text.

- *Thank you for your feedback. Some sections of the results have been re-written [see lines 326-327, 335, 343, 349-351]*

11. Table 2. The “total” row is not necessary. Please delete. Please re-order the category of staff according to the number from the largest to the smallest.

- *Thank you for your feedback. Corrections have been made accordingly. [see lines 243-244]*

12. Chart 4. Should explain and describe how the data in chart 4 was calculated, in terms of “summative” – which the term is only appeared and described in the discussion (one time) and conclusion (one time).

- *Thank you for your feedback. Chart 4 has been explained and described as ‘The MHD VF of each indicator was summative of all “recounted” and “reported” data points from the fifteen selected health facilities. Also, the VF of the overall malaria indicator was summative of all “recounted” and “reported” data points for the key indicators (Number of suspected malaria cases, Number of suspected malaria cases tested, Number of malaria cases tested positive, Number of OPD malaria cases treated with ACTs from the fifteen selected health facilities’ [see lines 356-360]*

Discussion and conclusions

13. Discussion: Please add more literature to compare and describe what are the difference in lessons learnt with other studies. The comparative of writing about other literature is weak in the discussion session.

- *Thank you for your feedback. Literature regarding the selected key indicators for the study is scanty for comparative writing about other studies. However, we have managed to do a few more to enrich our discussions. [see lines 399-401]*

14. Please discuss the point that VF values for the indicators (summative), except OPD cases treated with ACT, were within the acceptable range, but they all lied below “1” which is over-reported. Please explain and discuss for this point, which could tend to be more over-reported in the reality or other health facilities not included in the study.

- *Thank you for your feedback. Our interpretation is consistent with the definitions provided in the methods section. Please refer to Table 2 [i.e. % of facilities that* ***over-report*** *by more than 10% (VF < 0.90, % of facilities that* ***under-report*** *by more than 10% (VF > 1.10)]*

15. Conclusion: pg 14, line 6, it said malaria “**eradication”.** Is it truly that the program aimed for malaria **eradication** or **elimination**? Please recheck.

- *Thank you for your feedback. The error has been corrected to “elimination”. [line 466]*

16. The conclusion should be more precise and concise, touching all key findings and recommendation for program implementation. There is a duplication of mentioning about the “OPD cases treated with ACT” and suggest to combine the duplicate description. Please revise the conclusion session.

- *Thank you for your feedback. The conclusion has been revised to incorporate your feedback. [lines ….]*

17. Recommendation must be training, refresher training and strengthen regular supervision & monitoring plan by the national program. Further research is required, but only focus on the OPD cases and ACT treatment indicator is not the key recommendation from this paper. Yes, it is the only one indicator which needs improvement and pay attention, but the recommendation should be more in general and benefit for the malaria program as an overview. Please revise the recommendation.

- *Thank you for your feedback. The conclusion has been revised to incorporate your feedback. [lines 453-473]*

Thank you for your reconsideration and re-evaluation prior to consideration for publication. My colleagues and I appreciate your time and effort and look forward to hearing from you.

Sincerely,

Richard Okyere Boadu (PhD)

Department of Health Information Management
School of Allied Health Sciences

College of Health and Allied Health Sciences

University of Cape Coast

Cape Coast, Ghana

[richard.boadu@ucc.edu.gh](mailto:richard.boadu@ucc.edu.gh)
