# Supplementary material for: Assessment of health staff’s proficiency and quality of key malaria indicators in rural district of Ghana
Source: PLoS One. 2022 Oct 27;17(10):e0274700. doi: 10.1371/journal.pone.0274700 (PMC9612565; doi:10.1371/journal.pone.0274700)
Supplement: Attachment — Submitted filename: Reviewer comment_PONE-D-22-00045R1_20June2022.docx [file pone.0274700.s004.docx]

**Reviewer comment for PLOS ONE**

Date of review: 20 June 2022

Manuscript Number: PONE-D-22-00045R1

Article Type: Research Article

Full Title: Assessment of health staff’s proficiency and quality of key malaria indicators in Rural District of Ghana

Corresponding Author: Richard Okyere Boadu, University of Cape Coast, Cape Coast, GHANA

**Reviewer comment**

Summary of the research and overall impression

The comments were addressed and revised accordingly. However, there are still required to improve in results and discussion sessions, in terms of technical data presentation and English language editing. I would strongly recommend to consult/ take the proper English language editing service for academic publication writing and language of data presentation throughout the manuscript.

Discussion on specific area of improvement

1. Ethical consideration: pls mention the name of institution that the approval was issued, together with approval number. For example: Ethical approval was obtained from xxxx institution (approval number xxxx).

Results

2. Majority of the description in the text for the table and figures are still not revised yet. Pls describe and interpret the key findings ONLY in the text, not to duplicate information with the table and text. Pls revise all text in the result session and check the use of past-tense (verb) consistently in the result session in terms of English language editing.

For example: “*The specialization of respondents included: Doctor (2.0%), Pharmacist (2.0%),*

*252 Physician Assistant (2.0%), Nurses/Midwives (42.0%), Technical Officers (14.0%), Dispensing Technician (2.0%),253 Health Information Officers/ Biostatisticians (8.0%), and Lab scientist/Technician (6.0%) as shown in Table 3.”* [need to revise, this is just description of the result and duplicated with the information from the table. This is not the interpretation of the key findings.]

The above sentence should be described as followed as the example. *“The majority of the respondents were nurses/ midwives (42.0%), followed by the technical officers (14.0%) and health information officers/ biostatisticians (8.0%). Doctors and physician assistants were contributed only 2% of the respondents respectively.”*

Discussion

3. Thank you for revising, yet, discussion is still needed to improve more concise in description in writing. I would recommend to consult/ take the proper English language editing service for academic writing.

Recommendation

I would like to propose the **major revision** for this manuscript.
